# Three perspectives on learning in a simulated patient scenario: a qualitative interview study with student, simulated patient, and teacher

**DOI:** 10.1186/s41077-023-00249-0

**Published:** 2023-03-20

**Authors:** Sten Erici, Daniel Lindqvist, Mats B. Lindström, Christina Gummesson

**Affiliations:** 1 Centre for Teaching and Learning, Faculty of Medicine, Department of Clinical Sciences Malmö, Lund University, Malmö, Sweden; 2 Department of Clinical Sciences Lund, Psychiatry, Faculty of Medicine, Lund University, Lund, Sweden; 3 Office for Psychiatry and Habilitation, Psychiatry Research Skåne, Lund, Sweden; 4 Department of Clinical Sciences Malmö, Psychiatry, Faculty of Medicine, Lund University, Malmö, Sweden; 5 Faculty of Medicine, Department of Clinical Sciences Malmö, Lund University, Malmö, Sweden

**Keywords:** Student learning, Communication skills training, Professional identity formation, Content analysis, Simulated patients

## Abstract

**Introduction:**

Patient simulation can be useful for medical students in developing communication skills for vulnerable situations. Three participants are primarily involved in the patient simulation activities: the student, the simulated patient (SP), and the teacher. We here aimed to explore these participants’ perceptions of learning in a patient simulation scenario.

**Methods:**

We conducted individual interviews with eight students, three teachers, and one SP at a psychiatry placement of a Medical Doctor Program (5th year). During the interviews we asked the participants to watch a video of their participation in a patient simulation session. Thus, we obtained three perspectives on each of the eight recordings. We analysed our data with qualitative content analysis.

**Results:**

Three themes were generated: identity formation, collaborative learning, and learning intentions. This highly emotional scenario forced students out of their comfort zone, to the intersection of their roles as private person and professional. The SP perceived the collaborative creation of the scenario as significant learning. The teacher took a professional position and perceived the learning in the perspective of a future colleague.

**Conclusions:**

The mutually created emotionally loaded scenario was found to be important from all three perspectives, forcing the students to identify unexpected ways of communicating.

This possibly enhanced their professional identity development. Implications for future research can be to explore the process of skills transfer.

## Background

Simulated patients (SP) are a preferred option for helping medical students develop advanced communication skills [1–5]. The SP model may be particularly useful for developing communication skills required for complex and vulnerable situations such as in psychiatric patient encounters [6], and the use of SP may allow for more authentic practice in a safe environment.

A formative SP activity can be divided into two parts: scenario and feedback. The scenario is created by the teachers and the SP-trainer based on a case and tailored to the intended learning outcomes [3, 4]. The scenario is the hands-on part of the simulation, where students gain the experiences that will be discussed during the feedback. The feedback is central for reflection and learning [7, 8]. There are usually at least three active participants in an SP activity: student, SP, and teacher. There may also be additional participants, such as observing students, but they usually play a more passive role in the creation of the scenario.

Previous studies have indicated that students describe their learning experiences of a single SP activity as positive; they also typically report high satisfaction [9, 10].

### The active student

One objective for medical students is to develop competencies in their role as communicator, as described in competency frameworks such as CanMEDS (Canadian Medical Education Directives for Specialists) [11]. To facilitate the development of communicative competencies, the student should take an active role in their encounter with the SP. Previous research indicates that optimal learning takes place when students actively take part in the simulated interaction [12] and the simulated conditions are close to authentic [13–15]. Authentic simulations are considered to reflect actual workplace learning situations, which are important for students in developing their professional identity and confidence in various clinical situations [1, 3]. O’Reilly et al. [14] suggested that a high level of authenticity supports students in reflecting on their dual roles as private persons and medical professionals.

### The simulated patient

As noted above, authenticity is considered central for student learning during a simulation [9, 13, 16, 17]. Thus, one major task for the SP is to create scenarios perceived as authentic for the student [4, 9, 16, 18]. The SP needs to be a flexible communicative partner to individualize the communication [2, 9, 16, 19–21]. Bell and Kozlowski [22] described the importance of individualizing training to meet the needs of the learner and better support their self-regulatory learning processes. It is also beneficial if the SP has the intended learning outcomes clear [3, 4, 23], as the SP is expected to merge the synopsis with the student’s communicative behaviour and learning outcomes, in an authentic, flexible way [4, 9, 16, 19].

### The teacher

Teachers perform a dual role in workplace learning: as Dornan et al. states [24], ‘*An effective workplace teacher is someone who can simultaneously support students and challenge them in a way that builds practical competence and a positive state of mind.’*

The authenticity of an SP scenario is influenced by the teachers’ pre-brief before the scenario. Muckler et al. [25] stresses the importance of the pre-brief in preparing students for the fidelity of the simulation as well as achieving the intended learning outcomes. If done well, students sign an immaterial ‘fiction contract’ before entering the scenario [25], which is meant to motivate them to suspend their disbelief about unrealistic aspects of a simulation and engage with the scenario as if it was real. The teachers are important in supporting the creation of the fiction contract.

These three active participants—student, SP, and teacher—are all potential contributors to the mutual creation of learning in a scenario part of an SP activity. To our knowledge, only a few studies [9, 21] have investigated factors influencing perceived learning during an SP-scenario from the perspectives of all three active participants. There is thus a need to explore these factors more thoroughly.

In the current study, we aim to obtain further understanding of an SP scenario on vulnerable communicative situations in psychiatry with high emotional impact. We investigated which factors promoted learning by examining the SP scenario from perspectives of all the three active participants. This can aid in the development of more effective learning activities on challenging patient communication.

## Method

### Study design

For this study, we adopt a social constructivist paradigm [26], which assumes that reality and knowledge is constructed through human activity and interaction. Researchers utilizing this paradigm therefore attend to the active co-construction of knowledge during interactions between all participants. To perform this study, we used a qualitive approach based on data from interviews. Moreover, the researcher and object of study are interactively linked. The first author organized the SP activity and thereby had an in-depth understanding of the studied activity prior to the study. The research group was composed of specialists in medical education and psychiatry and had extensive experience on qualitative approach, although the main author was less experienced in the field.

### Study setting

This study took place at Lund University, in the south of Sweden, focusing on the psychiatric rotation of the Medical Education Programme. The curriculum covers patient communication skills during all eleven semesters. These skills are taught through lectures, seminars, roleplay with peers, work-based learning, and, at the time of the study, one SP activity.

The simulated case used was a patient in acute crisis [27] with suicidal features, tailored to the learning outcomes. A professional actor was enrolled as SP and thoroughly prepared for the simulation. During the psychiatric placement, students were divided into groups of four, overseen by a teacher (a physician from a psychiatric clinic). We asked all participants their permission to video record the SP activities; all gave their approval.

The scenarios with the feedback in action, during the simulation, was recorded. The feedback on action, after the simulation, was excluded.

### Data collection and analysis

Three researchers were involved in this study: the main author (doctoral student) and two senior medical education researchers.

### Participants

We used convenience sampling for data collection. Students in semester 9 were recruited. Thirty-two students took part in the SP activity. Eight volunteered to participate in the study and were interviewed after the session. None had prior experience of SP activities.

Three teachers and the SP were also interviewed. Two of the teachers were psychiatry residents and one was a physician specialised in psychiatry. The teachers had some previous experience in working with SPs. The SP had little experience in working in patient simulation outside this project but had extensive experience in improvisational acting and a particular interest in acting for educational purposes.

### Data collection

#### Interviews

**Fig. 1:**
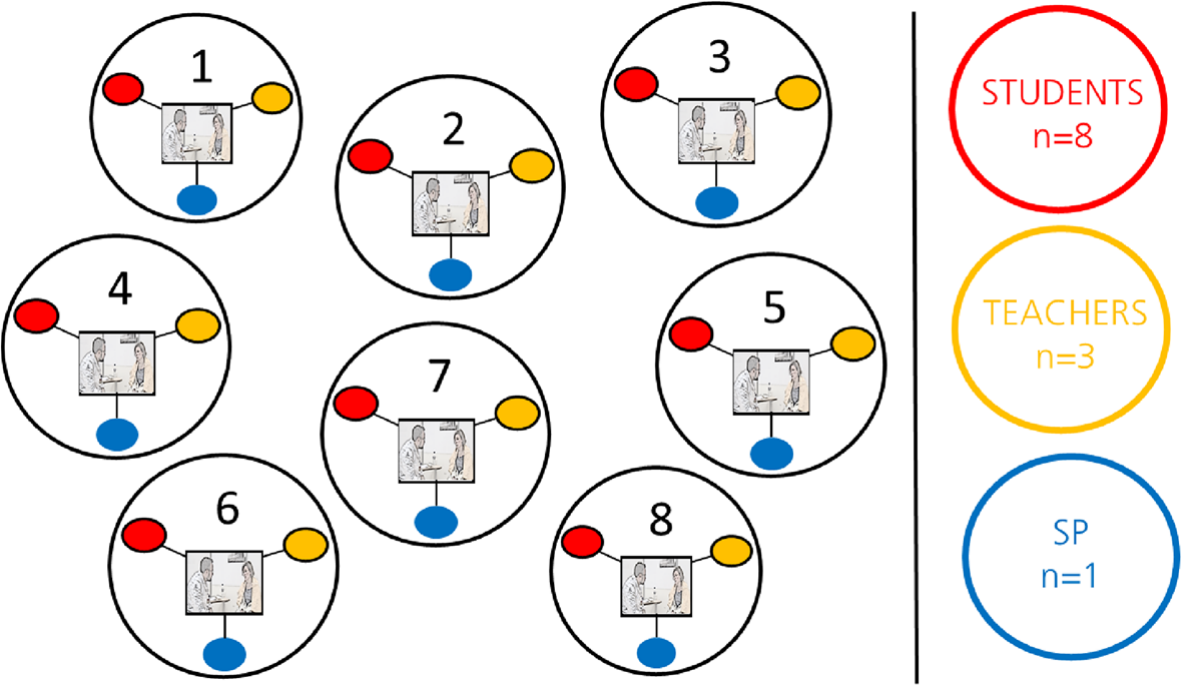
Eight simulated scenarios created 24 interviews with different participants. Participants watched video(s) of their own simulated scenario

The interviews were semi structured following interview guides created for each different role of participants, i.e., student, SP or teacher. When interviewing we used stimulated recall technique [28] utilizing video recordings of the scenarios to support memory and get another perspective of the simulation. The interview guides were tailored for the stimulated recall to trigger the participants’ narration on the perceived learning. One-on-one interviews with students and teachers were performed within four days after the SP activity. Interviews with the SP were performed within 2 weeks after the simulated event. Twenty-four interviews in total were conducted on 8 recordings with different participants, the first author and a senior researcher performed all interviews. Each student participated in one interview, the SP in eight and the teachers participated in one to three interviews (Fig. 1).

All participants were informed that they were going to watch recordings of their own simulated encounters and describe if and how any learning took place among students. They were told that they, or the interviewer, could stop and start the recording at any time to answer questions or to elaborate. Each interview lasted about 20 min. All interviews were audio recorded. A third party transcribed the interviews verbatim. The main author checked the transcripts against the recordings for accuracy.

#### Analysis

The interviews with the students, the SP and the teachers were defined as separate perspectives and the analysing process was applied on each perspective.

**Fig. 2:**
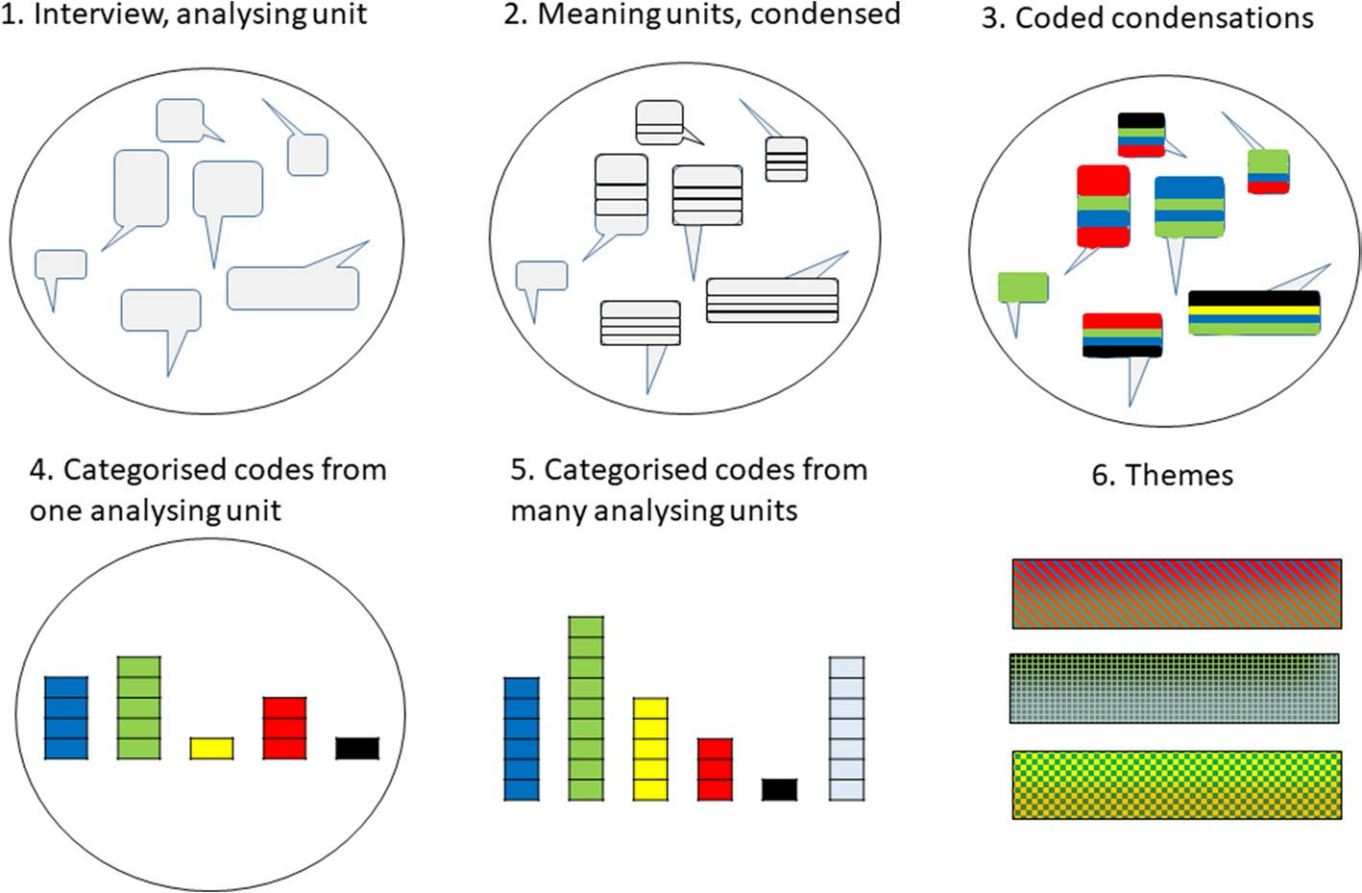
The analyzing process was performed on one perspective at a time. Step 1–5 was based on the manifest content. Step 6 was developed from the latent content

Inductive content analysis was used [29]. We treated each interview as one analysis unit during the first four steps of the content analysis (Fig. 2). Subsequently, we clustered the material into larger major units of analysis, including different interviews from the same perspective. The first author read each interview several times to obtain an overall sense of the content and to identify distinct meaning units. These meaning units were condensed and then abstracted into codes, which in turn were grouped into categories from which themes were generated (Fig. 2). The analysis was discussed in an iterative process among the researchers. The analysis was based on the manifest content until the step of generating themes. The creation of themes was performed on the latent level of the content (Tables 1 and 2). First subthemes were generated through discussions among the researchers, from these subthemes main themes were generated in the final step. The process of abstracting the material was achieved through multiple discussions among the research group to reach an agreement.

**Table 1:** Example of the abstraction process from identified meaning units to themes

Manifest level				Latent level	
Meaning unit	Condensed meaning unit	Code	Category	Subtheme	Theme
In this situation, you sense ‘which is my role here?’, ‘what is my task?’. It is not the same as if I would have been a fellow human, but you slowly work around it and get that understanding. It doesn’t help if I confirm or I can confirm but I cannot solve anything. The only thing I can do is to affirm, ‘it sounds very hard’	The student reflects on the role he/she has in the interaction	The physician	The professional role vs the private affects the contact	Awareness of the professional and private role	Identity formation

**Table 2:** Results of content analysis, organized by themes (in bold), with subthemes, categories and supporting quotes

Subtheme	Category	Example quotes
**Identity formation.** ***‘Necessity is the mother of invention’***		
Being conscious of the two roles: as a professional and a private person	The role of the physician	‘Yes, a little, because you become like that again—now she is very upset and then, the problematic dilemma is how much I should comfort because I still want her trust, well you want to build trust but it should not be too much outside the doctors' role’ (S)
		‘You become in a way very curious as a person and you want to cave down a bit in the yummy bits though it is maybe not the right way to behave as a professional’ (S)
Being surprised in a safe atmosphere makes the students lower the guard and be more present	Manage emotional responses	‘Just as I said, you can observe your own responses to unexpected events in the consultation’ (S)
The simulation was positive for learning	Exposed to strong emotions	‘Yes, it is probably some sort of reaction of fear. A little bit, anyway. Thus, learning …’ (S)
A certain amount of uncertainty creates learning	Insecurity supports learning	‘Balanced stress, balanced surprise, but being safe in that you can get help when you need it. I think that is a rather optimal learning curve if you consider how we live our life, yes but also if we consider how children learn and all other things, you should have the right amount and there has to be someone to consult if it breaks down’ (T)
**Collaborative learning.** ***‘It takes two to tango’***		
It was easy to be on two levels simultaneously, one fictive and one real	Easy to put oneself in a situation	‘You sign a contract -Now we are going to roleplay’ (S)
It takes two to tango	Actions of the SP	‘And as a patient now […..] that perspective it is nothing you… what can I say… regulate, you are on some sort of meta level but the basis, the emotions and the relation you cannot adjust, well you can but what happens is based on genuine responses, experienced responses of how you were treated’ (SP)
A detailed structure supports the opportunity for learning	The interactive design of the simulation	‘Well, it becomes more or less varied, and it depends on their [students’] responses and how they grasp things’ (SP)
**Learning intentions.** ***‘A path on the map shows the way forward’***		
To be pushed to discard one’s planned communication but keep the communicative strategies was a learning experience	Planned communication	‘I think many of the students have a long list in mind on what they have to ask. That is what we as teachers also have talked much about. You have to do a suicidal risk assessment; you have to ask if they have children…. well, there are some mandatory questions’ (T)

*S* student, *T* teacher, *SP* simulated patient

#### Trustworthiness

We used an inductive approach with a focus on uncovering different perspectives on students’ perceived learning during the SP activity. The first author mainly conducted the analysis. The first author also served as coordinator of the SP activities and thus was deeply involved in these activities. The other members of the research team balanced the first author’s position, being unfamiliar with the SP methodology but having thorough knowledge of medical education. One researcher conducted the interviews and one contributed with an outside perspective in the analysis. All researchers met regularly during the process. The composition of the research team made it possible to discuss the different perspectives and reach a consensus on the analysis.

The original statements were traceable through the entire analysis process but anonymised. The researchers could trace the statements from quote to theme but could not connect the condensed meaning units with a specific interview or interviewee. All the informants participated under regular working and study circumstances, and they told the interviewer that they had positive feelings about the SP activity.

## Results

Three major themes were generated:

1. Identity formation: ‘*Necessity is the mother of invention.’*
2. Collaborative learning:* ‘It takes two to tango.’*
3. Learning intentions: *‘A path on the map shows the way forward.’*

### Identity formation

‘As my private self, I really just want to say to the patient, ‘Cry!’ But now I'm a doctor so I want her to talk.’ (S [Student])

This theme originated from the emotional load in the scenario, which forced students to reflect on which role they were adopting: a private person offering comfort or a professional physician. As a teacher states about a student: ‘And how do you meet someone who is suffering in a professional role and not in the comforting, caretaking role…he really has to work on that.’ (T [Teacher])

This unexpected communicative situation pushed students into the intersection between these roles. They had to invent new strategies to engage in professional patient communication, describing their learning as occurring within the conflict between these two roles*.* The students engaged in meta-level reasoning about this emotionally strained situation, particularly how it was linked to the development of their professional role.

‘Yes, it is probably some sort of reaction of fear. A little bit, anyway. Thus, learning.’ (S)

Being surprised supported identity formation – in other words, their use of unexpected communicative strategies expanded their views of themselves. They could discuss their role as professional doctors from different angles. Students reflected on the learning as the result of encountering an unexpected event in a safe situation which provided them with clear feedback afterwards. They described this learning as intense.

The teachers played a crucial role in preparing the students for the exercise and motivating their signing of the ‘fiction contract’. The students noted that the learning conditions of the activity were to a large extent supported by the teachers’ preparations.

### Collaborative learning

‘It becomes more or less varied, and it depends on their attitude and how they approach the communication.’ (SP)

Students’ perceived learning emerged through their interactions with the SP. As one student expresses the learning process: ‘Being able to take notice on own emotions, and reactions on what the SP did and how she was or how the conversation proceeded.’ (S)

The SP and the student mutually created these learning experiences. The SP described sensing a learning space between them, where both they and students acted and responded. The SP described that she had a scripted synopsis to relate to but was dependent on the student’s contribution in creating the learning experiences. ‘And as a patient now […..] that perspective it is nothing you… what can I say… regulate, you are on some sort of meta level but the basis, the emotions and the relation you cannot adjust.’ (SP)

One subtheme was the emotional load that the SP regulated in collaboration with the student. The SP expressed this load as existing on two levels: present in the immediate situation which required clear responses but also in the ‘background’ and influenced by the script. With support from the script, the SP established an emotional load to put students in their zone of proximal development.

### Learning intentions

‘Can he make sure that will keep that he will become a good doctor!’ (T)

The teachers’ perspectives on learning in the SP scenario focused on students’ future professional role and what they should do to be a good doctor.

‘I think many of the students have a long list in mind on what they have to ask. That is what we as teachers also have talked much about. You have to do a suicidal risk assessment; you have to ask if they have children…. well, there are some mandatory questions.’ (T)

The teachers took an assessment-oriented perspective on the students’ performances, describing the performances as what to do and not do as a professional doctor.

## Discussion

An important finding in this study was the perceived impact on learning when students were forced to see themselves from the outside in their role as a professional physician. Moreover, the mutual creation of heightened emotions in the simulation by the SP and the student were perceived as important for learning. All three groups of participants said that authenticity and emotional load were important factors for learning, which is consistent with previous studies [9, 14, 25]. The emotionally loaded scenario forced students to identify new ways of communicating, which may have enhanced their professional identity development.

Rowe et al. similarly noted the importance of challenging emotional tasks that relate to real life in maximizing ownership of student learning [30]. The ongoing internal negotiations between the professional and personal self were also noted by the students in the current study. This sense of ownership over one’s development may provide a foundation for students’ professional identity formation. As McNaughton [31] expresses it: ‘At the heart of the practice of medicine is the dialogical relationship between doctor and patient. Tending to human suffering requires mastery of sophisticated technical and clinical skills and nuanced negotiations of self and other on a number of fronts (both personal and professional)’. Much of the students’ perceived learning takes place at the intersection of these roles, the private person offering comfort and the professional physician [16, 32]. It is important for professional identity development to balance between empathy and efficiency in practice which is shown in our study and also by Chew et al. [32].

The teachers had a crucial role in preparing students for the scenario. In line with Muckler et al. [13], they paved the way for the students to suspend their disbelief. The collaboration was mainly between the student and the SP, who mutually created the ‘learning space’. The SP kept the learning outcomes in mind, but both students and SP were dependent on each other’s input. This finding supports results from previous studies where a high level of adaptive learning [22] and students’ level of influence on the activity [12] have been suggested to affect learning in a positive way.

The teachers had a two folded perspective, on one hand they supported the actual scenario and on the other hand they assessed the student as a potential future colleague. Bearman et al. [33] indicates that clinical teachers may view assessment and facilitating learning as separate endeavours. It could be that the teachers took a perspective in far future distance from the scenario. But on the other hand, it may support the students’ long-term professional identity formation and it is on the teachers’ responsibility to assess the students’ communication.

One of the aims for the SP was to direct the student by regulating their actions so that students persist in their individual zone of proximal development (ZPD). As described by Groot et al. [17], taking the simulation outside students’ ZPD may impede their learning. However, ‘a simulation at the far edge of the ZPD causes constructive friction leading to an experience that generates a motivation to learn’ [17].

In their collaboration, the SP takes the role of a ‘more knowledgeable other’. This role is emphasized in Vygotsky’s social cultural theory, described by Tolsgaard et al. [34] as important for simulation-based learning. The knowledgeable other can push the student into their ZPD [34] which is what the SP in our study strived for by regulating their communicative actions to balance the emotional load.

The long-term development of the professional role and the self-assurance in the students may be supported by the simulation activities. However, achieving learning of communicative skills transferring to other educational contexts seems to be challenging. Shariff et al. [35] described that there are difficulties for sustaining and transferring learning from simulations over time and across different situations. As elements of communication skills are generic, it would be desirable for elements of the simulated activity to be recognized as useful in other learning situations. We intend to investigate the issue of transfer to other educational contexts further. In the future, it would be interesting to further explore the transfer of skills to other communicative learning situations than highly emotional conditions. It is also important how the learning in patient simulations is integrated in other parts of the medical education.

The students were in their fifth year of their MD programme, but it was the first SP activity they encountered. This could be a limitation and may explain their perspective on identity formation and their surprise at the SP’s actions. It is possible they were quite comfortable in their physician role but were still excited of the SP event. As Hanna and Fins [36] notes, overuse of simulation can have a negative effect—it may teach students to perform based on the expectations of the SP and the teacher, which can lower the authenticity of the situations. In this way, they become more simulacra of physicians rather than real ones. It seems to be important to keep a sense of novelty and uniqueness salient.

### Limitations

This study has some limitations. Observing peers were present in the simulation. However, they were not included in the present study. They may have influenced the scenario during feedback in action, which could have influenced participants’ actions. By getting feedback from peers the active student might have been inspired to go further in their zone of proximal development and explore other communicative actions then he/she would have done on his/her own. On the other hand, the observing peers can put pressure on the active student to perform in certain way to show communicative competency which could be unfavourable for learning. In future research, it would be interesting to explore the observer perspective. How does an active guide-on-the-side student influence and perceive learning during a simulated scenario?

Participant selection was done by inviting students to volunteer. There is a risk that only those with positive attitudes toward the use of SPs agreed to be interviewed. However, our intention was to explore their experiences and thoughts, which should capture both positive and negative feelings about the session.

The first author was involved in multiple areas of the study, which could influence the interpretations. We strived to balance that by holding frequent discussions within the research team throughout the analysis process.

## Conclusions

In the SP activities, informants described the formation of a professional physician identity through the support of the adaptive collaborative learning space with emotionally loaded cases. The mutual creation of learning by the student and the SP was an important feature for active and self-regulated learning.

## Data Availability

The datasets used and/or analyzed during the current study are available from the corresponding author on reasonable request.

## Abbreviations

SP: Simulated patient
CanMEDS: Canadian Medical Education Directives for Specialists
S: Student
T: Teacher
MD: Medical doctor
ZDP: Zone of Proximal Development
